# 2,3,5,6-Tetra­fluoro-1,4-bis­({[(5-methyl­thio­phen-2-yl)methyl­idene]amino}­meth­yl)benzene

**DOI:** 10.1107/S1600536814011398

**Published:** 2014-05-24

**Authors:** Hai-Kun Luo, Rui-Rui Qin, Ming-Yang He

**Affiliations:** a School of Petrochemical Engineering, Changzhou University, Changzhou 213164, People’s Republic of China

## Abstract

The title compound, C_20_H_16_F_4_N_2_S_2_, is a flexible bis­thio­phene-type Schiff base ligand with a perfluorinated backbone. The terminal thio­phene rings are almost normal to one another with a dihedral angle of 83.8 (2)°, and they are tilted to the central tetra­fluorinated benzene ring with dihedral angles of 61.2 (2) and 77.7 (1)°. In the crystal, there are π–π inter­actions involving the benzene ring and the thiophene ring of a symmetry-related molecule with a centroid–centroid separation of 3.699 (3) Å.

## Related literature   

For background information on thio­phene-based Schiff base ligands, see: Hee & Soon (2007 ▶); Fang *et al.* (2001 ▶). For related fluorine-functionalized complexes, see: Chen *et al.* (2012 ▶). For the synthesis of the title compound, see: Zhang *et al.* (2011 ▶).
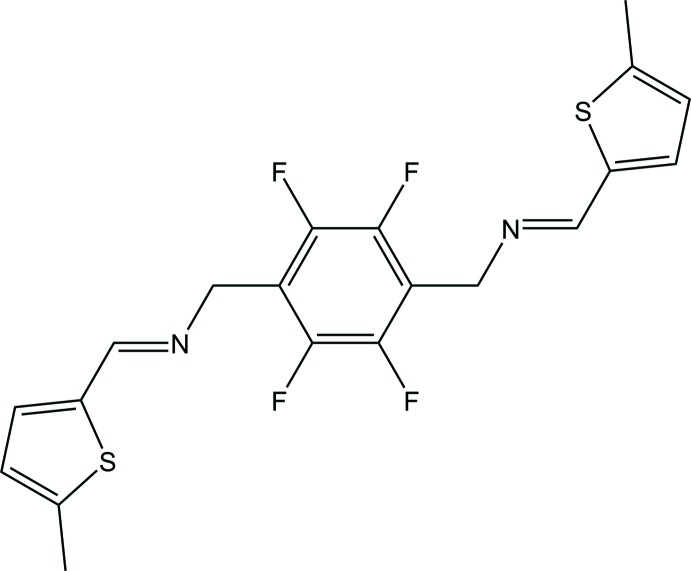


## Experimental   

### 

#### Crystal data  

C_20_H_16_F_4_N_2_S_2_
*M* *_r_* = 424.47
Monoclinic, 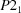

*a* = 9.472 (2) Å
*b* = 8.8083 (19) Å
*c* = 12.335 (3) Å
β = 111.459 (4)°
*V* = 957.8 (4) Å^3^
*Z* = 2
Mo *K*α radiation
μ = 0.32 mm^−1^
*T* = 296 K
0.28 × 0.22 × 0.20 mm

#### Data collection  

Bruker APEXII CCD diffractometer
Absorption correction: multi-scan (*SADABS*; Bruker, 2007 ▶) *T*_min_ = 0.914, *T*_max_ = 0.920
5721 measured reflections
3286 independent reflections
2897 reflections with *I* > 2σ(*I*)
*R*_int_ = 0.039

#### Refinement  

*R*[*F* ^2^ > 2σ(*F* ^2^)] = 0.048
*wR*(*F* ^2^) = 0.129
*S* = 1.06
3286 reflections
255 parameters
1 restraint
H-atom parameters constrained
Δρ_max_ = 0.45 e Å^−3^
Δρ_min_ = −0.29 e Å^−3^

### 

Data collection: *APEX2* (Bruker, 2007 ▶); cell refinement: *APEX2* and *SAINT* (Bruker, 2007 ▶); data reduction: *SAINT*; program(s) used to solve structure: *SHELXS2013* (Sheldrick, 2008 ▶); program(s) used to refine structure: *SHELXL2013* (Sheldrick, 2008 ▶); molecular graphics: *SHELXTL* (Sheldrick, 2008 ▶) and *DIAMOND* (Brandenburg, 2005 ▶); software used to prepare material for publication: *SHELXTL*.

## Supplementary Material

Crystal structure: contains datablock(s) I, global. DOI: 10.1107/S1600536814011398/bh2498sup1.cif

Structure factors: contains datablock(s) I. DOI: 10.1107/S1600536814011398/bh2498Isup2.hkl

Supporting information file. DOI: 10.1107/S1600536814011398/bh2498Isup3.cml

CCDC reference: 1003658

Additional supporting information:  crystallographic information; 3D view; checkCIF report

## Figures and Tables

**Fig. 1.**
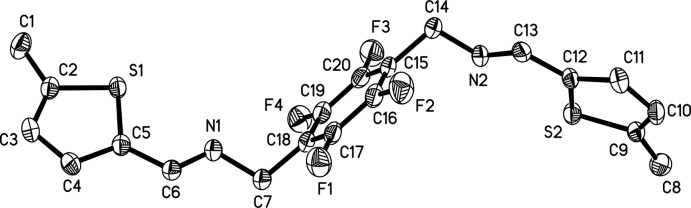
Molecular structure of the title compound with displacement ellipsoids drawn at the 30% probability level. Hydrogen atoms are omitted for clarity.

**Fig. 2.**
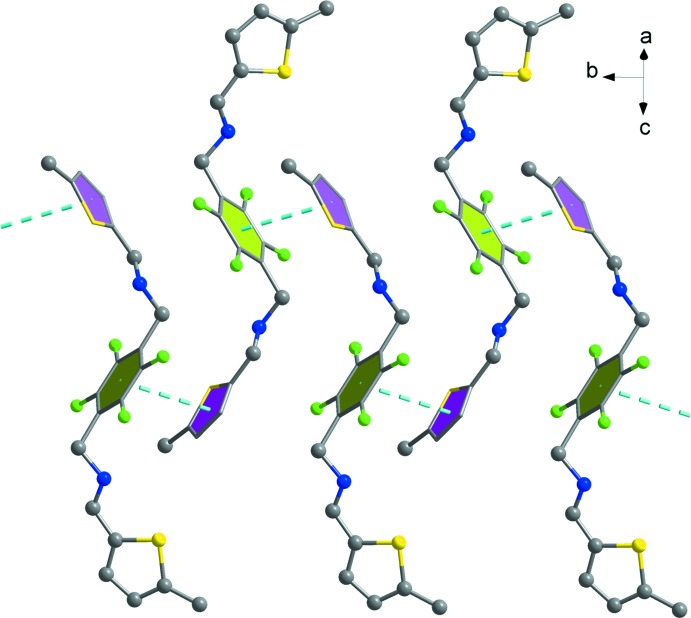
View of the supramolecular chain structure constructed via intermolecular π–π interactions (hydrogen atoms are omitted for clarity).

## supplementary crystallographic information

### 2.1. Synthesis and crystallization

The title compound was synthesized and purified according to the method described in Zhang *et al.* (2011), through a condensation of 2,3,5,6-tetra­fluoro-1,4-benzene­dimethanamine with 5-methyl­thio­phene-2-carboxaldehyde (yied 86%). ^1^H NMR (CDCl_3_): δ 2.42 (s, 6H, CH_3_), 2.76 (s, 4H, CH_2_), 6.73 (s, 2H, CH), 7.15 (s, 2H, CH), 7.90 (s, 2H, CH). HRMS: [M—H]^-^ calculated: 424.48, measured: 424.13. Colourless needle-like single crystals (m.p. 475.3-475.9 K) suitable for X-ray analysis were obtained by dissolving the compound (20 mg) in di­chloro­methane (6 ml), which was then slowly evaporated at room temperature over a period of about one week.

### 2.2. Refinement

Crystal data, data collection and structure refinement details are summarized in Table 1. All H atoms bound to C atoms were assigned to calculated positions, with C—H = 0.97 (methyl­ene), 0.96 (methyl), and 0.93 Å (aromatic), and refined using a riding model, with *U*_iso_(H)=1.5*U*_eq_ (carrier C) for the methyl groups and *U*_iso_(H)=1.2*U*_eq_ (carrier C) for other H atoms.

### 3. Results and discussion

In the past decade, thio­phene based bidentate Schiff base ligands have been utilized intensively to assemble various coordination compounds with intriguing structural features and potential applications (Hee & Soon, 2007; Fang *et al.*, 2001). As part of our ongoing studies of the fluorine-substituted effect on the crystal structures of coordination polymers (Chen *et al.*, 2012), herein, we wish to report the crystal structure of the title compound.

A perspective view of the compound, including the atomic numbering scheme, is shown in Fig. 1. X-ray diffraction study demonstrates that the compound crystallizes in the monoclinic space group *P*2_1_ with the molecule placed in general position. The bond lengths and angles are within normal ranges. The terminal thio­phene rings are almost perpendicular to each other, with a dihedral angle of 83.8 (2)°, and they are inclined to the tetra­fluorinated benzene ring with dihedral angles of 61.2 (2) and 77.7 (1)°, respectively. The crystal structure (Fig. 2) includes π–π inter­actions where π systems are separated by 3.699 (3) Å

### Crystal data

**Table:**

C_20_H_16_F_4_N_2_S_2_	*D*_x_ = 1.472 Mg m^−^^3^
*M**_r_* = 424.47	Melting point: 475 K
Monoclinic, *P*2_1_	Mo *K*α radiation, λ = 0.71073 Å
*a* = 9.472 (2) Å	Cell parameters from 2369 reflections
*b* = 8.8083 (19) Å	θ = 2.3–24.9°
*c* = 12.335 (3) Å	µ = 0.32 mm^−^^1^
β = 111.459 (4)°	*T* = 296 K
*V* = 957.8 (4) Å^3^	Needle, colourless
*Z* = 2	0.28 × 0.22 × 0.20 mm
*F*(000) = 436	

### Data collection

**Table:**

Bruker APEXII CCD diffractometer	3286 independent reflections
Radiation source: fine-focus sealed tube	2897 reflections with *I* > 2σ(*I*)
Graphite monochromator	*R*_int_ = 0.039
φ and ω scans	θ_max_ = 26.2°, θ_min_ = 1.8°
Absorption correction: multi-scan (*SADABS*; Bruker, 2007)	*h* = −11→11
*T*_min_ = 0.914, *T*_max_ = 0.920	*k* = −10→10
5721 measured reflections	*l* = −14→15

### Refinement

**Table:**

Refinement on *F*^2^	Primary atom site location: structure-invariant direct methods
Least-squares matrix: full	Secondary atom site location: difference Fourier map
*R*[*F*^2^ > 2σ(*F*^2^)] = 0.048	Hydrogen site location: inferred from neighbouring sites
*wR*(*F*^2^) = 0.129	H-atom parameters constrained
*S* = 1.06	*w* = 1/[σ^2^(*F*_o_^2^) + (0.0803*P*)^2^] where *P* = (*F*_o_^2^ + 2*F*_c_^2^)/3
3286 reflections	(Δ/σ)_max_ < 0.001
255 parameters	Δρ_max_ = 0.45 e Å^−^^3^
1 restraint	Δρ_min_ = −0.29 e Å^−^^3^
0 constraints	

### Fractional atomic coordinates and isotropic or equivalent isotropic displacement parameters (Å^2^)

**Table:**

	*x*	*y*	*z*	*U*_iso_*/*U*_eq_	
C1	0.3718 (7)	−0.0116 (7)	−0.2492 (5)	0.0715 (16)	
H1A	0.4443	−0.0092	−0.2869	0.107*	
H1B	0.2720	−0.0269	−0.3064	0.107*	
H1C	0.3961	−0.0933	−0.1938	0.107*	
C2	0.3765 (5)	0.1358 (6)	−0.1875 (4)	0.0487 (11)	
C3	0.4580 (6)	0.2597 (7)	−0.1859 (5)	0.0641 (14)	
H3A	0.5235	0.2671	−0.2264	0.077*	
C4	0.4360 (6)	0.3784 (6)	−0.1174 (5)	0.0639 (14)	
H4A	0.4844	0.4719	−0.1088	0.077*	
C5	0.3365 (5)	0.3421 (6)	−0.0649 (4)	0.0456 (10)	
C6	0.2812 (5)	0.4377 (6)	0.0058 (3)	0.0444 (9)	
H6A	0.3222	0.5341	0.0263	0.053*	
C7	0.1268 (7)	0.5008 (6)	0.1089 (4)	0.0572 (12)	
H7A	0.0365	0.5530	0.0585	0.069*	
H7B	0.2050	0.5759	0.1440	0.069*	
C8	−0.4832 (7)	0.6390 (8)	0.6706 (6)	0.0723 (15)	
H8A	−0.4703	0.6929	0.7412	0.108*	
H8B	−0.5734	0.5782	0.6489	0.108*	
H8C	−0.4920	0.7103	0.6096	0.108*	
C9	−0.3507 (5)	0.5399 (5)	0.6891 (4)	0.0484 (11)	
C10	−0.2339 (6)	0.5088 (6)	0.7870 (4)	0.0540 (12)	
H10A	−0.2252	0.5487	0.8590	0.065*	
C11	−0.1253 (5)	0.4109 (6)	0.7725 (4)	0.0520 (12)	
H11A	−0.0390	0.3779	0.8332	0.062*	
C12	−0.1609 (5)	0.3697 (5)	0.6588 (4)	0.0432 (10)	
C13	−0.0723 (5)	0.2770 (6)	0.6103 (4)	0.0490 (10)	
H13A	0.0170	0.2332	0.6605	0.059*	
C14	−0.0123 (7)	0.1595 (8)	0.4638 (5)	0.0677 (15)	
H14A	−0.0627	0.0651	0.4311	0.081*	
H14B	0.0796	0.1355	0.5294	0.081*	
C15	0.0264 (6)	0.2471 (6)	0.3727 (4)	0.0512 (11)	
C16	0.1508 (5)	0.3399 (6)	0.4012 (4)	0.0514 (11)	
C17	0.1834 (5)	0.4222 (6)	0.3173 (4)	0.0484 (11)	
C18	0.0924 (5)	0.4168 (5)	0.2023 (4)	0.0470 (10)	
C19	−0.0323 (5)	0.3238 (6)	0.1732 (4)	0.0523 (11)	
C20	−0.0650 (6)	0.2415 (6)	0.2566 (5)	0.0540 (12)	
F1	0.3086 (3)	0.5090 (4)	0.3519 (3)	0.0717 (9)	
F2	0.2454 (4)	0.3518 (5)	0.5127 (2)	0.0799 (10)	
F3	−0.1886 (4)	0.1531 (5)	0.2219 (3)	0.0827 (10)	
F4	−0.1265 (4)	0.3120 (4)	0.0623 (2)	0.0773 (9)	
N1	0.1783 (5)	0.3929 (5)	0.0402 (3)	0.0526 (10)	
N2	−0.1121 (5)	0.2535 (5)	0.5023 (3)	0.0544 (10)	
S1	0.26914 (13)	0.16095 (14)	−0.10223 (10)	0.0494 (3)	
S2	−0.32916 (13)	0.44947 (15)	0.57216 (9)	0.0503 (3)	

### Atomic displacement parameters (Å^2^)

**Table:**

	*U*^11^	*U*^22^	*U*^33^	*U*^12^	*U*^13^	*U*^23^
C1	0.080 (4)	0.073 (4)	0.072 (3)	0.005 (3)	0.040 (3)	−0.020 (3)
C2	0.049 (2)	0.057 (3)	0.044 (2)	0.006 (2)	0.0217 (18)	−0.002 (2)
C3	0.072 (3)	0.066 (3)	0.076 (3)	0.001 (3)	0.053 (3)	0.005 (3)
C4	0.066 (3)	0.053 (3)	0.094 (4)	−0.012 (2)	0.054 (3)	−0.002 (3)
C5	0.047 (2)	0.051 (3)	0.043 (2)	0.002 (2)	0.0208 (18)	0.000 (2)
C6	0.048 (2)	0.045 (2)	0.043 (2)	0.0020 (19)	0.0193 (17)	0.000 (2)
C7	0.084 (3)	0.049 (3)	0.053 (3)	0.011 (2)	0.041 (3)	0.004 (2)
C8	0.083 (4)	0.063 (4)	0.092 (4)	0.017 (3)	0.057 (3)	0.009 (3)
C9	0.058 (3)	0.046 (3)	0.054 (3)	−0.005 (2)	0.036 (2)	−0.002 (2)
C10	0.065 (3)	0.064 (3)	0.045 (2)	−0.011 (2)	0.033 (2)	−0.010 (2)
C11	0.050 (2)	0.070 (3)	0.038 (2)	−0.003 (2)	0.0185 (18)	0.001 (2)
C12	0.049 (2)	0.047 (2)	0.043 (2)	−0.0045 (18)	0.0288 (18)	−0.0011 (19)
C13	0.055 (3)	0.048 (3)	0.053 (3)	0.007 (2)	0.031 (2)	0.009 (2)
C14	0.098 (4)	0.055 (3)	0.078 (4)	0.026 (3)	0.065 (3)	0.011 (3)
C15	0.066 (3)	0.047 (3)	0.059 (3)	0.012 (2)	0.043 (2)	0.001 (2)
C16	0.055 (3)	0.060 (3)	0.043 (2)	0.015 (2)	0.024 (2)	−0.001 (2)
C17	0.049 (2)	0.052 (3)	0.054 (2)	0.006 (2)	0.029 (2)	−0.007 (2)
C18	0.054 (2)	0.047 (3)	0.050 (2)	0.0107 (19)	0.031 (2)	−0.002 (2)
C19	0.057 (3)	0.056 (3)	0.047 (2)	0.007 (2)	0.023 (2)	−0.007 (2)
C20	0.057 (3)	0.046 (3)	0.070 (3)	0.000 (2)	0.037 (2)	−0.011 (2)
F1	0.0589 (16)	0.086 (2)	0.074 (2)	−0.0124 (16)	0.0292 (15)	−0.0083 (18)
F2	0.085 (2)	0.105 (3)	0.0476 (16)	0.012 (2)	0.0216 (14)	0.0005 (17)
F3	0.081 (2)	0.080 (2)	0.101 (2)	−0.028 (2)	0.0487 (19)	−0.018 (2)
F4	0.084 (2)	0.089 (2)	0.0508 (16)	−0.0067 (18)	0.0163 (14)	−0.0123 (17)
N1	0.069 (2)	0.052 (2)	0.047 (2)	0.0029 (18)	0.0333 (19)	−0.0021 (18)
N2	0.068 (3)	0.054 (2)	0.059 (2)	0.008 (2)	0.044 (2)	0.005 (2)
S1	0.0526 (6)	0.0510 (6)	0.0553 (6)	−0.0051 (5)	0.0325 (5)	−0.0047 (6)
S2	0.0544 (6)	0.0589 (7)	0.0404 (5)	0.0066 (5)	0.0205 (4)	−0.0004 (5)

### Geometric parameters (Å, º)

**Table:**

C1—C2	1.498 (7)	C9—S2	1.724 (4)
C1—H1A	0.9600	C10—C11	1.403 (7)
C1—H1B	0.9600	C10—H10A	0.9300
C1—H1C	0.9600	C11—C12	1.366 (6)
C2—C3	1.333 (8)	C11—H11A	0.9300
C2—S1	1.724 (4)	C12—C13	1.447 (6)
C3—C4	1.407 (8)	C12—S2	1.712 (5)
C3—H3A	0.9300	C13—N2	1.262 (6)
C4—C5	1.362 (6)	C13—H13A	0.9300
C4—H4A	0.9300	C14—N2	1.461 (6)
C5—C6	1.441 (6)	C14—C15	1.515 (7)
C5—S1	1.718 (5)	C14—H14A	0.9700
C6—N1	1.260 (6)	C14—H14B	0.9700
C6—H6A	0.9300	C15—C16	1.371 (7)
C7—N1	1.470 (6)	C15—C20	1.377 (7)
C7—C18	1.502 (6)	C16—F2	1.343 (6)
C7—H7A	0.9700	C16—C17	1.388 (7)
C7—H7B	0.9700	C17—F1	1.342 (6)
C8—C9	1.476 (8)	C17—C18	1.364 (7)
C8—H8A	0.9600	C18—C19	1.373 (7)
C8—H8B	0.9600	C19—F4	1.336 (6)
C8—H8C	0.9600	C19—C20	1.382 (7)
C9—C10	1.334 (7)	C20—F3	1.339 (6)
			
C2—C1—H1A	109.5	C11—C10—H10A	122.7
C2—C1—H1B	109.5	C12—C11—C10	112.1 (4)
H1A—C1—H1B	109.5	C12—C11—H11A	123.9
C2—C1—H1C	109.5	C10—C11—H11A	123.9
H1A—C1—H1C	109.5	C11—C12—C13	127.7 (4)
H1B—C1—H1C	109.5	C11—C12—S2	110.8 (3)
C3—C2—C1	128.8 (4)	C13—C12—S2	121.4 (3)
C3—C2—S1	110.7 (4)	N2—C13—C12	122.3 (5)
C1—C2—S1	120.5 (4)	N2—C13—H13A	118.8
C2—C3—C4	113.9 (4)	C12—C13—H13A	118.8
C2—C3—H3A	123.1	N2—C14—C15	108.3 (4)
C4—C3—H3A	123.1	N2—C14—H14A	110.0
C5—C4—C3	113.1 (5)	C15—C14—H14A	110.0
C5—C4—H4A	123.5	N2—C14—H14B	110.0
C3—C4—H4A	123.5	C15—C14—H14B	110.0
C4—C5—C6	128.2 (5)	H14A—C14—H14B	108.4
C4—C5—S1	110.1 (4)	C16—C15—C20	116.3 (4)
C6—C5—S1	121.6 (3)	C16—C15—C14	122.1 (5)
N1—C6—C5	121.2 (4)	C20—C15—C14	121.6 (5)
N1—C6—H6A	119.4	F2—C16—C15	119.9 (4)
C5—C6—H6A	119.4	F2—C16—C17	118.4 (4)
N1—C7—C18	109.6 (4)	C15—C16—C17	121.7 (4)
N1—C7—H7A	109.8	F1—C17—C18	120.0 (4)
C18—C7—H7A	109.8	F1—C17—C16	118.1 (4)
N1—C7—H7B	109.8	C18—C17—C16	121.9 (4)
C18—C7—H7B	109.8	C17—C18—C19	116.6 (4)
H7A—C7—H7B	108.2	C17—C18—C7	123.3 (5)
C9—C8—H8A	109.5	C19—C18—C7	120.1 (4)
C9—C8—H8B	109.5	F4—C19—C18	120.1 (4)
H8A—C8—H8B	109.5	F4—C19—C20	118.3 (5)
C9—C8—H8C	109.5	C18—C19—C20	121.6 (4)
H8A—C8—H8C	109.5	F3—C20—C15	119.7 (5)
H8B—C8—H8C	109.5	F3—C20—C19	118.4 (5)
C10—C9—C8	129.6 (5)	C15—C20—C19	121.9 (5)
C10—C9—S2	110.5 (4)	C6—N1—C7	116.9 (4)
C8—C9—S2	119.9 (4)	C13—N2—C14	117.2 (4)
C9—C10—C11	114.5 (4)	C5—S1—C2	92.2 (2)
C9—C10—H10A	122.7	C12—S2—C9	92.0 (2)
